# Reactions for actions? Trust in protective behaviors and safeguarding measures in the early phase of the Covid-19 pandemic in Sweden

**DOI:** 10.1016/j.pmedr.2023.102133

**Published:** 2023-02-09

**Authors:** Elin M. Andersson, Margareta Norberg

**Affiliations:** a Department of Psychology, Umeå University, Umeå, Sweden; b Department of Epidemiology and Global Health, Umeå University, Umeå, Sweden

**Keywords:** COVID-19, Response efficacy, Strategy efficacy, Prevention & control, Protective behaviors, Safeguarding measures, Sweden, Health behavior

## Abstract

To minimize the spread of Covid-19, changing every-day behavior has been key. Trust in the effectiveness of individual protective measures (response efficacy) and confidence in collective safeguarding measures (strategy efficacy), offers an incitement for acting adequately. Efficacy beliefs of protective measures might be especially relevant to study in the Swedish context, since Sweden, in contrast to countries facing hard lock-downs, launched safeguarding measures based on individual responsibility and voluntary actions. We aimed to assess associations between on the one hand, response efficacy and strategy efficacy, and on the other hand, propensity for behavior change and support of protective measures. Furthermore, to assess associations between the efficacy beliefs and comprehension of and confidence in information about the virus, prosocial beliefs and worry of Covid-19. Reactions were assessed in a Swedish sample close in time to experiences via the SEMA^3^ app from March 25th to May 17th 2020. Study participants had replied to questions on strategy efficacy (n = 175) or response efficacy (n = 157) and 146 participants had replied to both. High response efficacy was associated with propensity for behavior change, support of protective measures and confidence in Covid-19 information. Low strategy efficacy was associated with lower comprehension of and confidence in information about Covid-19. The results suggest that strengthening efficacy beliefs can be a way to promote protective behaviors. Furthermore, the result underscores the importance of information being easy to understand and trustworthy. Finding ways to increase public understanding of the effectiveness of protective measures, including vaccination, seems crucial in pandemic times.

## 1 Introduction

The pandemic Covid-19 has demanded adaptation of individuals and societies. Changing every-day behavior to minimize the spread of the virus has been key. In this context, *response efficacy,* trust in the effectiveness of individual protective measures (Rogers, 1975, Maddux and Rogers, 1983), and *strategy efficacy,* confidence in collective safeguarding measures, offers an incitement for acting adequately. Compared to health threats from non-communicable diseases, where efficacy beliefs regarding personal agency are important for health behaviors, during a pandemic, confidence in the effectiveness of collective safeguarding measures and trust in decision makers’ strategy to manage the spread and of the virus are also essential. This phenomenon has been described also as collective efficacy, here we refer to it as *strategy efficacy*.

Results from studies assessing the predictive power of threat and coping appraisals have found that, in a variety of health- and safety-related contexts, response efficacy and self-efficacy, are the strongest predictors for intentions and behaviors (Milne et al., 2000, Norman et al., 2005). Thus, individuals’ perceived severity or susceptibility of Covid-19 might be weaker motivators for adherence to restrictions. Risk communication messages stating that some groups are at higher risk are vital to target interventions, yet such messages might have negative effects (Balog-Way and McComas, 2020). Relaxed attitudes among low-risk groups can contribute to increasing the spread of the virus. It has also been found that when uptake of protection behaviors was low despite high levels of knowledge about Covid-19, willingness to restrict one's everyday life was higher if motivated to protect vulnerable others (Betsch, 2020). In line with this, it has been suggested that vaccination should be framed as a prosocial behavior (Hong and Hashimoto, 2021).

Research of determinants of protective behaviors during previous pandemics shows that being a woman, having high education and possibly also being of higher age were associated with behavior modification. Furthermore, beliefs in the effectiveness of recommended behaviors, perceived susceptibility to and severity of the disease, trust in authorities and satisfaction with communication about the disease was also associated with compliance with protective behaviors (Bish and Michie, 2010).

Efficacy beliefs of protective measures might be especially relevant to study in the Swedish context, since Sweden, in contrast to countries with hard lock-downs during the early phase of the pandemic, launched safeguarding measures based mainly on individual responsibility. In particular, citizens were expected to voluntarily practice social distancing to contribute to *flatten the curve* (referring to graphs showing new cases) for the sake of the health care system and the work load of health care workers. In a study of risk perception of Covid-19 around the world, personal efficacy was of particular importance in Sweden, compared to other countries. Collective efficacy (strategy efficacy) was on the other hand far less important, contrary to other countries, for example Japan and the US (Dryhurst et al., 2020).

### 1.1 Covid-19 strategy in Sweden

It could be argued that the Swedish way of launching safeguarding measures, strongly emphasizing individual responsibility, differed from other countries. At least, the Swedish policy was questioned and intensively debated. In national and international media, it was suggested that the population was “exposed to an experiment”. On the other hand, in the end of April 2020, the Swedish policy was also described by WHO as “a role model” (Miltimore, 2020).

It is outside the scope of this paper to compare different strategies of managing the pandemic. However, for understanding of the study setting, it should be noted that in the Swedish governance, non-political expert authorities play an important role. Consequently, The Public Health Agency of Sweden was leading the way not only by monitoring the development, but also by informing the public about restrictions aiming to reduce the spread of the virus and gave recommendations to the government about actions to take. At the time for the data collection, citizens received daily information and instructions for individual protective behaviors at press conferences held by the authorities. Expectations on individuals to take responsibility, show solidarity and follow recommendations and regulations was strong (Nygren and Olofsson, 2020). Some formal restrictions, such as online teaching for senior high schools and universities, and prohibition of visits to homes for the elderly were implemented during this early phase of the pandemic. However, shops and restaurants were open, and the messages from the authorities and the government repeatedly focused on the importance of all citizens taking part to reduce the spreading of the virus. For example: *Hold on, carry on, keep distance. Be part of the solution. Protect yourself and others from the infection. Change your behavior.*

### 1.2 Aim of the study

The aim of the study was to assess associations between on the one hand, response efficacy and strategy efficacy, and on the other hand, propensity for behavior change and support of protective measures. Furthermore, to assess associations between the efficacy beliefs and comprehension of and confidence in information about the virus, prosocial beliefs and worry of Covid-19.

## 2 Method

Information about the study was spread in social media, a local newspaper, by email to students at the Department of Psychology, Umeå University, Sweden and by approaching an organization for retired people. Inclusion criteria were (1) age 18 + years old, (2) Swedish speaking, (3) having access to a smartphone. Reactions were assessed with the SEMA^3^ app (Smartphone Ecological Momentary Assessment), a software for intensive survey research using smartphones (Koval et al., 2019), from March 25th to May 17th 2020. A code was assigned to each participant, but no code-key linking the code to the participant’s name, phone number or mail address exists. The data collection has previously been described in detail (Schulz et al., 2021).

The data came from a longitudinal study, in which new questions where added during the data collection period, in order to closely monitor the development in society. For the variables of interest in this present study, the suitability of a cross sectional analysis was tested with a sensitivity analysis. If individuals had replied to a question more than once, the first and last assessments were compared. For all variables, there was no statistically significant difference between timepoints, except for worry of Covid-19, which increased slightly over time (Schulz et al., 2021). Therefore, a cross sectional design was applied. In case of more than one reply to a question, the mean value was used. In this study, only participants who had received questions on strategy efficacy (n = 175) or response efficacy (n = 157) were included, and 146 had replied on both.

The questionnaire included questions also on propensity for behavior change, support of protective measures, worry about the virus, comprehension of and confidence in information about Covid-19 as well as pandemic effect on prosocial beliefs (see Appendix).

For response efficacy and strategy efficacy, associations to other variables were tested by comparing the lower tertile (low efficacy) and upper tertile (high efficacy) with T-test. The study was conducted in accordance with the Helsinki Declaration, GDPR legislation was followed, and participants provided informed consent.

## 3 Results

In the study population, 79.7 % were female, 88.1 % had university education or were university students. Among participants, 35.6 % were 20–44 years old, 49.0 % were 45–68 years, and 15.4 % were 70–82 years old. For response efficacy there was a trend, however not statistically significant, that when the sample was divided into tertiles, the proportion of females, participants with high education and of higher age was increasing with level of response efficacy. Furthermore, response efficacy tended to be lower among individuals living in big cities. Distribution of demographic factors in groups with low and high efficacy beliefs are presented in Table 1. Comparisons between groups with low versus high efficacy beliefs regarding attitudes, experiences and reactions to Covid-19 are presented in Table 2.

**Table 1:** Distribution of demographic factors in groups with low and high efficacy beliefs.

	n	Low response efficacy	High response efficacy	p	n	Low strategy efficacy	High strategy efficacy	p
Participants, total*	105	50	55		120	63	57	
								
**Sex** n (%)	96	48	48	0.346	108	57	51	0.609
Men		14 (58.3)	10 (41.7)			7 (46.7)	8 (53.3)	
Women		34 (47.2)	38 (52.8)			50 (53.8)	43 (46.2)	
								
**Age group** n (%)	96	48	48	0.432	108	58	51	0.075
20–44		20 (58.8)	14 (41.2)			24 (68.6)	11 (31.4)	
45–69		22 (45.8)	26 (54.2)			25 (45.5)	30 (54.5)	
70–82		6 (42.9)	8 (57.1)			8 (44.4)	10 (55.6)	
								
**Education** n (%)	96	48	48	0.064	108	58	51	0.013**
No university		9 (75)	3 (25)			9 (90.0)	1 (10.0)	
University		39 (46.4)	45 (53.6)			48 (49.0)	50 (51.0)	
								
**Residence** n (%)	95	48	47	0.078	108	58	51	**0.008**
Big city		10 (58.8)	7 (41.2)			14 (58.3)	10 (41.7)	
Medium size city		34 (54.8)	28 (45.2)			30 (43.5)	39 (56.5)	
Rural		4 (25.0)	12 (75.0)			13 (86.7)	2 (13.3)	
								

* Because of technical problems with the SEMA^3^ app when participants received the first survey, there are some missing data on demographic variables.

** For assessment of education and strategy efficacy, Fishers exact test was used.

**Table 2:** Comparisons between groups with low versus high efficacy beliefs regarding attitudes, experiences and reactions to Covid-19.

	n	Low response efficacy	High response efficacy	p	n	Low strategy efficacy	High strategy efficacy	p
**Propensity for behavior change** M (SD)	95	45	50		115	61	54	
		6.45 (0.86)	6.89 (0.23)	**0.001**		6.86 (0.32)	6.51 (0.79)	**0.003**
								
**Support of protective measures** M (SD)	90	39	51		109	56	53	
		6.24 (0.94)	6.66 (0.44)	**0.013**		6.66 (0.46)	6.44 (0.75)	0.074
								
**Worry about Covid-19** M (SD)	105	50	55		119	62	57	
		6.12 (2.70)	6.50 (2.38)	0.450		7.64 (2.29)	5.89 (2.44)	**<0.001**
								
**News and press conferences** M (SD)	93	43	50		94	46	48	
Comprehension of information on Covid-19		5.72 (0.95)	6.16 (1.01)	**0.034**		5.78 (1.09)	6.30 (0.90)	**0.013**
Confidence in information on Covid-19		5.30 (1.41)	5.64 (1.24)	0.225		4.68 (1.53)	5.97 (0.99)	**<0.001**
								
**Pandemic related prosocial beliefs** M (SD)	105	50	55		118	61	57	**0.001**
		4.56 (0.93)	4.96 (1.26)	0.070		4.54 (1.18)	5.21 (0.94)	
								
**Strategy efficacy** M (SD)	98	45	53		115	*63*	*57*	
		4.52 (1.44)	5.11 (1.64)	0.065		*3.14 (1.06)*	*6.38 (0.40)*	***<0.001***
								
**Response efficacy** M (SD)	105	*50*	*55*		98	49	49	0.099
		*5.01 (0.51)*	*6.74 (0.25)*	***<0.001***		5.88 (0.78)	6.14 (0.77)	

## 4 Discussion

The overall aim of the study was to assess how trust in effectiveness of individual protective behaviors (response efficacy) and collective safeguarding measures (strategy efficacy) were related to attitudes, experiences and reactions to Covid-19. High response efficacy was associated with higher propensity for behavior change, higher support of protective measures and higher confidence in Covid-19 information. The positive association between response efficacy on one hand, and, on the other hand, propensity for behavior change and support of protective measures is an important finding, since this implies that strengthening response efficacy beliefs might be a way to promote protective behaviors. In line with our results, a study assessing behavior and worry at the start of the Covid-19 outbreak in UK, found that protective behaviours were associated with perceived effectiveness of individual behaviours, self-efficacy for engaging in these behaviours, greater worry and being more informed about Covid-19. Smith and colleges argue that, in the early phase of novel infectious disease outbreaks, communications should highlight the effectiveness of protective behaviours for reducing the spread of disease, and also highlight that behaviors can easily be adopted (Smith et al., 2022).

An Australian study, also undertaken during the early phase, found that even when level of risk was perceived as low, adopting avoidance behaviors such as cancelling social events or reducing ones’ use of public transport, was associated high perceived rating of effectiveness of behaviors and high level of perceived ability to adopt social distancing strategies (Seale et al., 2020). This is highly relevant, since this indicates the potential of strengthening efficacy beliefs in the general population, not least in groups who perceive low susceptibility and severity of disease. Adopting avoidance behaviours was also associated with high trust in authorities. In our Swedish sample, by contrast, even though high response efficacy was associated with higher propensity for behavior change and higher support of protective measures, at the same time, individuals with low strategy efficacy had higher propensity for behavior change. A possible interpretation is that study participants who doubted the effectiveness of safeguarding measures believed that a hard lock-down would be more effective. However, it should be pointed out that propensity for behavior change was high overall, also in the group with high strategy efficacy.

Participants with high response efficacy and participants with high strategy efficacy reported higher comprehension of information about Covid-19 and also higher confidence in information, although the difference in confidence in information between groups high and low in response efficacy was not statistically significant. Altogether, this emphasize the importance of messages from authorities being perceived as trustworthy and easy to understand, not least in an “infodemic” (The Lancet Infectious, 2020), where fake news competes with validated information. However, understanding the effectiveness of protective measures that focus on adopting avoidance behaviors, such as cancelling social events, might be a challenge. Individuals might need more support to understand the effect of refraining from things they want to do, because they will never see any evidence of the spread of infection that does *not* occur as a result of them making sacrifices. In this context, visual illustrations modeling the effect of social distancing on the spread of infection might facilitate the understanding of the effectiveness of avoidance behaviors (Nyqvist, 2020).

The finding that high strategy efficacy was associated with higher level of prosocial beliefs is consistent with the prompting messages by the authorities regarding the importance of all citizens taking part to reduce the spreading of the virus. For example: *Be part of the solution. Protect yourself and others from the infection.*

Understanding the effectiveness of protective behaviors might still be of importance, not least where vaccination coverage is low, but also if new mutations make the vaccines of today less effective. Monitoring and targeting efficacy beliefs regarding vaccines should also be highly relevant for increasing vaccination rates.

A limitation of the study was that the proportion of participants being female and having high education were large. Therefore, our results should be interpreted with caution.

A strength of the study was the inclusion of participants of all ages, important not at least since older individuals are at higher risk of contracting a severe Covid-19 infection. Another asset was the methodology of ecological momentary assessment which allowed participants to report their reactions close in time to experiences, which in turn would reduce the risk of recall bias. Also, since the collected data were anonymized, the risk of social desirability was minimal.

## 5 Conclusions

The result suggests that strengthening efficacy beliefs might promote protective behaviors during a pandemic. Furthermore, the result underscores the importance of information being easy to understand and trustworthy. Therefore, finding ways to increase public understanding of the effectiveness of protective measures, including vaccination, seems crucial in pandemic times.

## CRediT authorship contribution statement

**Elin M. Andersson:** Conceptualization, Data curation, Formal analysis, Investigation, Writing – original draft. **Margareta Norberg:** Conceptualization, Funding acquisition, Writing – review & editing.

## Declaration of Competing Interest

The authors declare that they have no known competing financial interests or personal relationships that could have appeared to influence the work reported in this paper.

## Data Availability

Data will be made available on request.

## Appendix – Variables

For variables where several questions were combined to capture the specific concept, the mean value of the included questions was calculated. All responses were given on Likert scales.

### Response efficacy

Three statements assessed response efficacy: (i) *Washing my hands frequently and carefully will protect me from getting infected, (*ii*) If I meet fewer people it will protect me from being infected* and (iii) *Keeping physically distance to people I meet will protect me from being infected.* The scale ranged from 1 = *Do not agree to* 7 = *Agree.*

### Strategy efficacy

Three different aspects of the Swedish strategy efficacy were assessed. (i) Sufficiency of protective measures: *Various measures have been taken to reduce the speed of the virus spreading, for example, the number of people allowed to be in a crowd is limited. When it comes to influencing how fast the corona virus is spreading, how effective do you think these resolved measures are?* The scale ranged from *1* = *I think the actions taken so far are insufficient,* to *7* = *I think the actions taken so far are good.* (ii) Belief in voluntary actions and individual responsibility: *Which strategy do you think works best when it comes to persuading people to follow guidelines for reducing the spread of infection, such as reducing their social contacts, over a prolonged period of time?* The scale ranged from 1 = *Monitoring people, laws and penalties* to 7 = *Advice, voluntary actions and individual responsibility*. (iii) Trust in the Swedish strategy: *What do you think of Sweden's strategy to limit the spread of the covid-19 infection?* The scale ranged from 1 = *Sweden should do more like other countries,* to *7* = *Sweden has a good strategy.*

### Propensity for behavior change

Participants were first asked: *What would you consider doing to protect yourself and others? Please tell us on a scale from 1* = *I would never do that) to 7* = *I would definitely do that.* Three statements were assessed: (i) *Cancel a holiday trip, (*ii*)Avoid crowded areas* and *(*iii*) Refrain from going to a restaurant or pub.*

### Support of protective measures

Participants were asked: *What would you consider to do to protect yourself and others? Please tell us on a scale from 1 (I am totally against that) to 7 (Have my full support).* Three items were assessed: (i) *Limited travel by air and train so that only absolutely necessary trips are made,* (ii) *Online education for high school and university* and (iii) *Visitors prohibited at elderly care homes.*

### Worry about corona virus

Worry of corona virus was assessed by the question: *To what extent are you worried about the corona virus?* Replies ranged from *0 Not worried at all* to *10 Very worried.*

### Comprehension of and confidence in corona information

Participants were asked *How do you experience news and/or press conferences on corona/covid-19?* and could reply on a scale ranging from *1* = *Difficult to understand* to *7* = *Easy to understand*. Confidence in information was assessed by the question *To what extent do you trust the information you receive through news and or press conferences?* Participants replied on a scale ranging from *1* = *Not at all* to *7* = *Completely.*

### Pandemic effect on prosocial beliefs

Perception regarding the effect of the pandemic on prosocial beliefs was assessed with the following question**:** *How do you think people are affected by the spreading of the corona virus?* with replies on a scale ranging from *1* = *The spread of the corona virus leads people to act primarily on their own interests* to 7 = *The spread of the corona virus causes people to stand up more for one another.*
